# Unique Properties of Hepatocarcinogenesis-Resistant DRH Rat Hepatocytes Linked or Not Linked to the *Drh1* Locus on Rat Chromosome 1

**DOI:** 10.4061/2011/424356

**Published:** 2011-07-25

**Authors:** Norikazu Hashimoto, Masahiro Yamamoto, Masaaki Miyakoshi, Hiroki Tanaka, Katsuhiro Ogawa

**Affiliations:** Department of Pathology, Section of Oncology, Asahikawa Medical College, 2-1-1-1 East, Midorigaoka, Asahikawa 078-8510, Japan

## Abstract

Hepatocarcinogenesis-resistant DRH rats exhibit few and small preneoplastic hepatocytic lesions during hepatocarcinogenesis, of which traits have been assigned to two major chromosomal regions, *Drh1* and *Drh2*. In this study, hepatocytes from DRH.F344-*Drh1*, a congenic strain in which the *Drh1* chromosomal region was replaced with that of F344 rats, were compared to hepatocytes from Donryu (original strain), DRH, and F344 rats. Although DRH hepatocytes exhibited low proliferation and p38 dephosphorylation after lead nitrate (LN) treatment despite cytokine and Cox2 activation, DRH.F344-*Drh1* hepatocytes exhibited high responses, as did Donryu and F344 hepatocytes. Moreover, although DRH hepatocytes were resistant to hepatotoxins, DRH.F344-*Drh1* hepatocytes were as sensitive to hepatotoxins as Donryu and F344 hepatocytes. However, DRH.F344-*Drh1* hepatocytes like DRH hepatocytes proliferated at lower rates *in vitro* and contained smaller nuclei than Donryu and F344 hepatocytes. Thus, low responses to LN and resistance to hepatotoxins in DRH hepatocytes were linked to the *Drh1* locus, while low proliferation *in vitro* and small nuclear size were not linked to the *Drh1* locus.

## 1. Introduction

DRH rats were established by the selective mating of Donryu rat progeny that exhibited resistance to hepatocarcinogenesis induced by the feeding of a 3′-methyl-4-dimethylaminoazobenzene (3′-Me-DAB) diet [1, 2]. DRH rats exhibit hepatocarcinogenesis resistance to 3′-Me-DAB as well as other hepatocarcinogens [3]. In DRH rats, preneoplastic hepatocytic lesions are smaller in size and fewer in number than those of ordinary rats [4, 5]. Genetic analysis has demonstrated that resistance is a dominant trait in a cross of DRH and Donryu rats [4] and that the number and size of preneoplastic hepatocytic lesions and the expression levels of glutathione-S-transferase-placental form (GST-P) mRNA and protein, a marker for preneoplastic hepatocytes, are strongly affected by highly significant quantitative trait loci (QTL) on rat chromosomes 1q (*Drh1*) and 4q (*Drh2*) [5, 6]. It has also been shown that the small size of preneoplastic hepatocytic lesions might be dependent mainly on the tissue environment; that is, transplanted DRH preneoplastic hepatocytes were observed to form large colonies in the livers of Donryu rats treated with dietary 2-acetylaminofluorene (2-AAF) plus partial hepatectomy (PH), which enables the selective growth of preneoplastic hepatocytes [7], and transplanted Donryu preneoplastic hepatocytes formed small colonies within the livers of DRH rats treated with the same protocol [8].

 DRH hepatocytes have a number of unique properties compared to hepatocytes of ordinary rats. First, DRH hepatocytes exhibit less proliferation after treatment with lead nitrate (LN) [4, 9], a nonnecrogenic hepatocyte proliferating agent [10], which is thought to be mediated, at least in part, by the cytokines generated by activated Kupffer cells [11, 12]. Second, DRH hepatocytes are resistant to various hepatotoxic chemicals, including carbon tetrachloride (CCl_4_), diethylnitrosamine (DEN), thioacetamide, 2-AAF, and 3′-Me-DAB [8]. Third, in primary culture, DRH hepatocytes exhibited less apoptosis and lower activation of p38 and Jun terminal kinase (JNK) than Donryu hepatocytes, although the expression levels of 8-hydroxyguanine and p53/p21 proteins that reflect DNA damage [13, 14] were comparable to those of Donryu hepatocytes [15]. Fourth, DRH hepatocytes are smaller in size than Donryu hepatocytes [15]. If some of these properties of DRH hepatocytes are linked to the* Drh1 *or *Drh2* locus, then the properties might also be related to the mechanism(s) of hepatocarcinogenesis resistance in DRH rats.

 DRH.F344-*Drh1* rats represent the congenic DRH strain in which the chromosome 1 segment that includes the *Drh1* locus is substituted with that of F344 rats [9]. These rats exhibit a high incidence of GST-P(+) foci in response to treatment with 3′-Me-DAB/PH and high liver cell proliferation after LN treatment, comparable to that in F344 rats, but formed intermediate-sized GST-P(+) foci and exhibited intermediate GST-P mRNA expression levels between the levels of F344 and those of DRH rats after treatment with DEN followed by dietary 3′-Me-DAB/PH [9]. This finding indicates that the *Drh1* locus contributes to the low incidence of GST-P(+) focus formation and the low level of liver cell proliferation in response to LN treatment, but it does not fully explain the smaller size of the GST-P(+) foci. In the present study, we investigated which properties of DRH hepatocytes are linked to the *Drh1* locus by comparing the hepatocytes of DRH.F344-*Drh1* rats with those of DRH, Donryu and F344 rats *in vivo* and *in vitro*. 

## 2. Materials and Methods

### 2.1. Animals and Treatments

**Figure 1:**
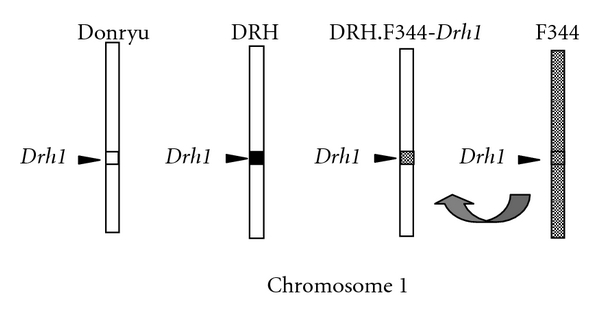
Schematic representation of the *Drh1* locus on chromosome 1 in Donryu, DRH, DRH.F344-*Drh1*, and F344 rats. In DRH.F344-*Drh1* rats, the region including the *Drh1 *locus is substituted with that of F344 rats.

Male 8- to 10-week-old DRH.F344-*Drh1* (kindly provided by Dr. Hiai, Medical Center of the Shiga Prefecture, Japan), DRH (Seak Yoshitomi, Fukuoka, Japan), Donryu (Charles River, Tokyo, Japan), and F344 rats (Charles River) were used. The *Drh1* locus on chromosome 1 of these rats is illustrated in Figure 1. The animals were maintained in 12 h light/dark cycles and given laboratory rodent pellet chow (Oriental, Tokyo, Japan) and water *ad libitum*. One group of rats was intravenously administered LN at a dose of 100 mmole/kg body weight, given bromodeoxyuridine (BrdU) on day 1, 2, or 3 after LN treatment, and sacrificed 1 h after BrdU treatment. The liver tissues were perfusion-fixed with a 10% buffered-formalin solution, immersed in fixative solution for 24 h, and then processed for paraffin embedding. The tissue sections were immunostained using an anti-BrdU antibody (Becton Dickinson, San Jose, Calif, USA), and the BrdU labeling index (LI) of hepatocytes was determined microscopically. Other groups of rats were administered DEN dissolved in physiological saline at a dose of 200 mg/kg body weight by intraperitoneal injection or CCl_4_ dissolved in olive oil at a dose of 5 mL/kg body weight by gastric tube and sacrificed 24 h later. Blood samples were taken from each rat, and serum was isolated to measure alanine aminotransferase (ALT) and aspartate aminotransferase (AST) levels. All animal procedures were approved by the Asahikawa Medical College committee according to the guidelines for humane care of laboratory animals and conducted according to the Asahikawa Medical College committee of animal care and use. 

### 2.2. Primary Hepatocyte Culture and Cell Proliferation Assay

Hepatocytes were isolated using a collagenase perfusion method [16] and plated on 12-well plates. They were first cultured in Williams' E medium with 10% fetal bovine serum (FBS) for 24 h and then cultured in hepatocyte growth medium (HGM) [17] supplemented with 5 U/mL penicillin, 100 *μ*g/mL streptomycin, and various concentrations of EGF (0, 1, 10, or 50 ng/mL) or HGF (0, 5, 20, or 100 ng/mL) for 3 days. The cultured hepatocytes were treated with [^3^H]-thymidine for 3 days after beginning culture with HGM, and [^3^H]-thymidine incorporation into DNA was determined using a liquid scintillation counter (Beckmann-*Coulter*, Palo Alto, Calif, USA). 

### 2.3. Nuclear Size Analysis

Freshly isolated hepatocytes were plated on a 15 mm diameter cover glass coated with Cellmatrix Type 1-A (Nitta Gelatin, Tokyo, Japan) in Williams' E medium supplemented with 10% FBS, cultured for 2 h to allow attachment to the cover glass, fixed in 1% buffered-formalin solution and stained by DAPI. The cells were observed under a fluorescence microscope (Nikon, Tokyo, Japan) equipped with a cool-scanning digital camera (Hamamatsu Photonics, Hamamatsu, Japan). Nonfluorescent and DAPI-stained nuclear images of hepatocytes were saved as Photoshop files, and each cell was divided into small single- or double-, medium single- or double- or large single- or double-nuclear cells using ImageJ software (National Institute of Health, Bethesda, Md, USA). At least 500 hepatocytes were measured for each rat, using three rats from each strain. 

### 2.4. Western Blot Analysis

Protein lysates were prepared from liver tissue samples, run on 8–13% polyacrylamide gels containing 0.1% sodium dodecyl sulfate and transferred to PVDF membranes (Amersham, Uppsala, Sweden). The membranes were probed with primary antibodies and then hybridized with horseradish peroxidase- (HRP-) conjugated anti-rabbit immunoglobulin (Amersham). The bound antibodies were detected using an ECL-plus kit (Amersham). 

### 2.5. Electromobility Shift Assay (EMSA)

EMSA was performed for nuclear extracts of LN-treated liver tissues, and the DNA-protein complexes were resolved on a 6% polyacrylamide gel. A biotin-labeled oligonucleotide representing an NF*κ*B consensus site (5′-AGTTGAGGGGACTTTCCCAGGC-3′) and a Stat3 consensus site (5′-TTCT GGG AAT T-3′) was used as a probe. 

### 2.6. Real-Time RT-PCR

Fresh liver samples were isolated at 0, 3, 6, 12, and 24 h after LN treatment, frozen in liquid nitrogen, and stored at −80°C until further use. RNA was isolated with Sepazol and reverse-transcribed using the generic oligo dT primer and reverse transcriptase (Invitrogen, Carlsbad, Calif, USA). The following PCR primers were used: for TNF-*α*, 5′-CCCATTTGGGAACTTCTCCT-3′ (forward) and 5′-AGATGTGGAACTGGCAGAGG-3′ (reverse); for IL-6, 5′-GCCCTTCAGGAACAGCTATG-3′ (forward) and 5′-TCAGTCCCAAGAAGGCAACT-3′ (reverse); for Cox2, 5′-GAGATACGTGTTGACGTCC-3′ (forward) and 5′-ACTGATGAGTGAAGTGCTGG-3′ (reverse). Real-time RT-PCR was performed using a LightCycler (Roche Molecular Biochemicals, Basel, Switzerland) with LightCycler3 Run software (version 5.32). Fluorescence was generated by a Platinum SYBR Green qPCR SuperMix UDG, and data were collected with LightCycler3 Data Analysis software (version 3.5.28). 

### 2.7. Statistical Analysis

Each experiment was performed at least three times, and representative data are shown. Data in bar graphs are given as the mean ± SD. The means were analyzed using Student's *t*-test or ANOVA, and *P* values less than 0.05 were considered statistically significant. 

## 3. Results

### 3.1. Hepatocyte Proliferation, Cytokine Activation, and p38 MAPK Hypophosphorylation after LN Treatment

**Figure 2:**
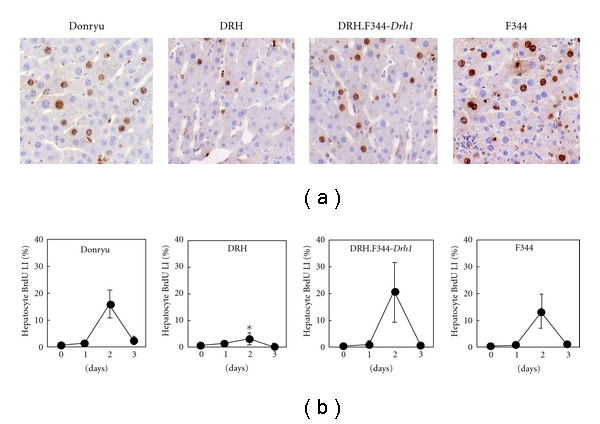
Proliferation of hepatocytes in Donryu, DRH, DRH.F344-*Drh1*, and F344 rats after LN treatment. (a) Day 2 after LN treatment. BrdU(+) hepatocyte nuclei occurred frequently in Donryu, DRH.F344-*Drh1*, and F344 livers but occurred much less frequently in DRH livers. (b) The BrdU LI in hepatocytes was most strongly increased on day 2 after LN treatment in Donryu, DRH.F344-*Drh1*, and F344 livers but was much lower in DRH livers. *P < 0.05 in comparison with DRH (*n* = 5) against Donryu (*n* = 5), DRH.F344-*Drh1* (*n* = 4), or F344 (*n* = 8).

**Figure 3:**
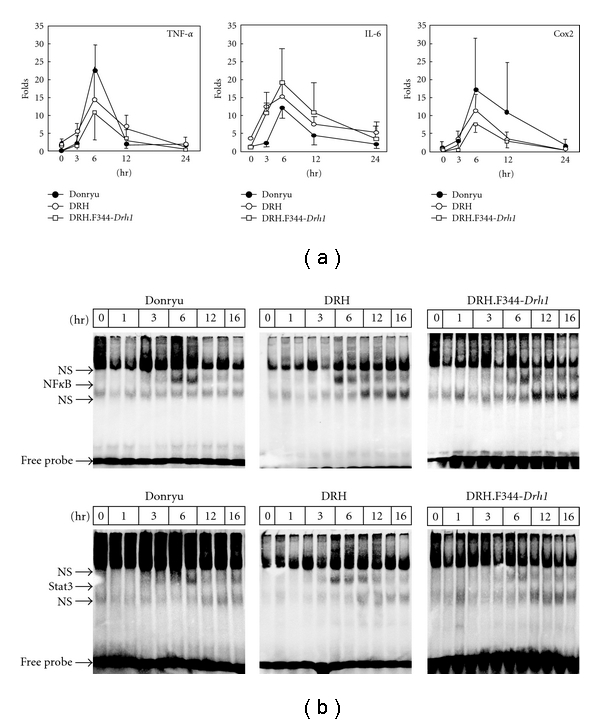
TNF-*α*, IL-6, and Cox2 mRNA expression levels and NF*κ*B/Stat3 activation after LN treatment. (a) Quantitative RT-PCR revealed that TNF-*α*, IL-6, and Cox2 mRNA expression levels peaked at 6 h after LN treatment in Donryu, DRH, and DRH.F344-*Drh1* livers. (b) EMSA for NF*κ*B (upper panel) and Stat3 (lower panel). Both NF*κ*B and Stat3 were most strongly activated at 6 h after LN treatment in Donryu, DRH, and DRH.F344-*Drh1* livers. NS: nonspecific bands.

When Donryu, DRH, DRH.F344-*Drh1*, and F344 rats were treated with LN, the BrdU LI markedly increased in DRH.F344-*Drh1* hepatocytes on day 2 as reported by Liu et al. [9], similarly to the increases observed in Donryu and F344 hepatocytes, but the BrdU LI did not increase in DRH hepatocytes (Figures 2(a) and 2(b)), indicating that the *Drh1* locus is linked to the low response of DRH hepatocytes to LN. It has been suggested that hepatocyte proliferation induced by LN is mediated at least partly by cytokines such as TNF-*α* and IL-6, which are generated by activated Kupffer cells [11, 12]. In addition, Cox2 protein expression in activated Kupffer cells, which leads to the overproduction of prostaglandins, has been shown to play an important role in hepatocyte proliferation [18]. Furthermore, when Donryu rats were treated with indomethacin, a Cox1/2 inhibitor, prior to and after LN treatment, the BrdU LI of hepatocytes decreased to approximately 50% of the values after LN treatment alone on day 2, indicating that prostaglandins at least partly mediate hepatocyte proliferation after LN treatment (unpublished data by M. Yamamoto). We therefore investigated whether the *Drh1* locus plays a role in cytokine and Cox2 activation after LN treatment. As shown in Figure 3(a), TNF-*α*, IL-6, and Cox2 mRNA expression levels were increased to the same extent from 3 to 12 h after LN treatment, peaking at 6 h in DRH.F344-*Drh1*, Donryu, and DRH livers, suggesting that the *Drh1* locus is not related to cytokine and Cox2 activation after LN treatment. Because TNF-*α* and IL-6 can activate NF*κ*B and Stat3, respectively, which are transcription factors that play an important role in hepatocyte proliferation [19], we investigated NF*κ*B/Stat3 activation by EMSA using nuclear lysates prepared from LN-treated livers and NF*κ*B/Stat3-specific oligonucleotide probes. Figure 3(b) illustrates that NF*κ*B/Stat3 activation occurred 6–16 h after LN treatment, with a peak at 6 h, in Donryu, DRH, and DRH.F344-*Drh1* livers.

**Figure 4:**
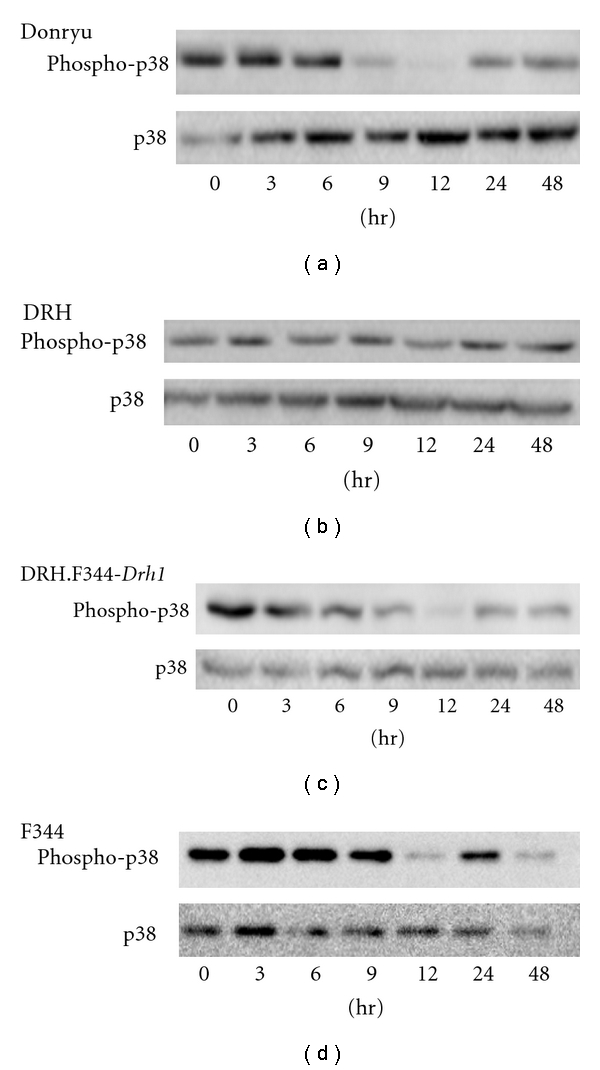
The phosphorylation status of p38 after LN treatment. Although p38 was hyperphosphorylated in normal livers of Donryu, DRH, DRH.F344-*Drh1*, and F344 rats, it was markedly hypophosphorylated in Donryu, DRH.F344-*Drh1*, and F344 livers at 12 h after LN treatment, but p38 hypophosphorylation occurred at much lower degrees in DRH livers.

 It has been reported that when the liver is exposed to toxic chemicals, the phosphorylation status of MAPK pathway proteins, including ERK1/2, JNK, and p38, fluctuates shortly after exposure [20–22]. We therefore investigated whether there was any difference in the phosphorylation status of MAPKs after LN treatment in the livers of Donryu, DRH, DRH.F344-*Drh1*, and F344 rats. ERK1/2, which was hypophosphorylated in normal livers, became hyperphosphorylated 3–12 h after LN treatment, whereas JNK, which was also hypophosphorylated in normal livers, showed no remarkable change in phosphorylation status after LN treatment in all rats (data not shown). However, although p38 was hyperphosphorylated in the normal livers of all Donryu, DRH, DRH.F344-*Drh1*, and F344 rats, as reported previously [20–22], it became hypophosphorylated after LN treatment in Donryu, DRH.F344-*Drh1*, and F344 livers, peaking at 12 h, but such hypophosphorylation was at much less degree in DRH livers (Figure 4). 

### 3.2. Susceptibility of Hepatocytes to Hepatotoxic Chemicals

**Figure 5:**
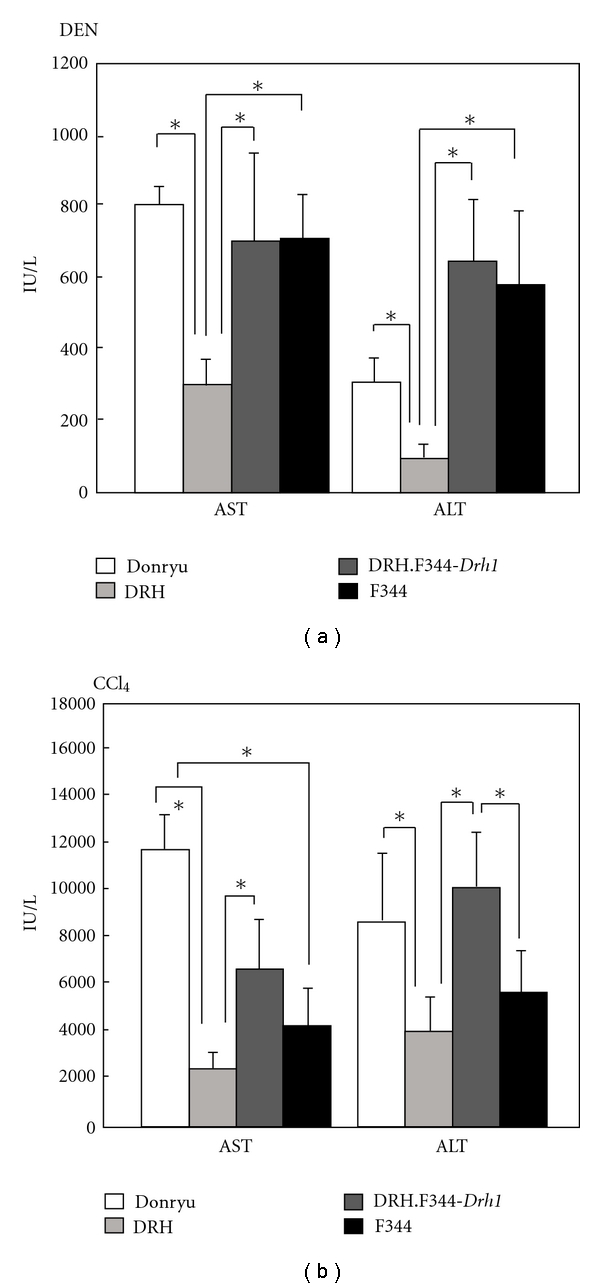
Liver injury after DEN and CCl_4_ treatment. (a) After DEN treatment, DRH.F344-*Drh1* rats (*n* = 6) exhibited high AST/ALT values, similar to those of Donryu (*n* = 6) and F344 rats (*n* = 5); these levels were much higher than those of DRH rats (*n* = 6). *P < 0.05. (b) After CCl_4_ treatment, DRH.F344-*Drh1 *rats (*n* = 10) also exhibited high AST/ALT values, similar to those of Donryu rats (*n* = 9); these levels were higher than those of DRH (*n* = 12) and F344 rats (*n* = 11). *P < 0.05.

DRH hepatocytes have been demonstrated to be resistant to various hepatotoxic chemicals, including CCl_4_, thioacetamide, DEN, 2-AAF, and 3′-Me-DAB [8]. To test the role of the *Drh1* locus in hepatotoxin susceptibility, Donryu, DRH, DRH.F344-*Drh1*, and F344 rats were administered 200 mg/kg DEN or 5 mL/kg CCl_4_ to compare liver injury 24 h after treatment. After DEN treatment, serum ALT and AST levels in DRH.F344-*Drh1* rats were as high as those in Donryu and F344 rats, while these levels were significantly lower in DRH rats (Figure 5(a)). After CCl_4_ treatment, DRH rats exhibited a lower degree of hepatic injury than Donryu rats as previously reported [8], while F344 rats also showed the lower degree of injury than Donryu rats (Figure 5(b)), presumably because the factor(s) other than the *Drh1* locus that was involved to the CCl_4_ susceptibility might be different between Donryu and F344 rats. However, DRH.F344-*Drh1* rats exhibited a higher degree of hepatic injury than DRH and F344 rats (Figure 5(b)).

### 3.3. Hepatocyte Proliferation * In Vitro*

**Figure 6:**
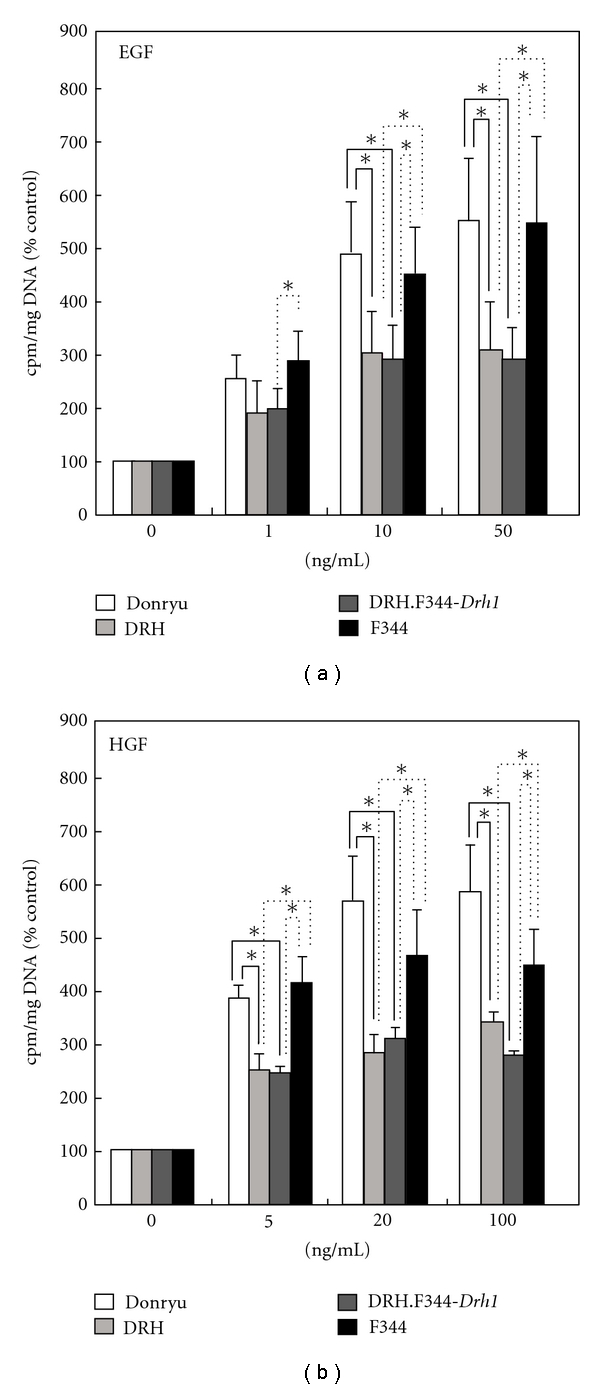
Uptake of [^3^H]-thymidine into hepatocyte DNA after EGF or HGF treatment. Although [^3^H]-thymidine uptake was increased after either EGF (a) or HGF (b) treatment, it was significantly lower in DRH and DRH.F344-*Drh1* hepatocytes than in Donryu and F344 hepatocytes (*P < 0.05).

We next addressed whether the *Drh1* locus plays a role in the proliferative capacity of hepatocytes. For this purpose, hepatocytes were isolated from Donryu, DRH, DRH.F344-*Drh1*, and F344 rats and cultured in the presence of various concentrations of EGF (0, 1, 10, or 50 ng/mL) or HGF (0, 5, 20, or 100 ng/mL). Because [^3^H]-thymidine uptake increased beginning on day 1 after growth factor treatment and peaked on day 3 in the hepatocytes of all rats (data not shown), the cells were then incubated with [^3^H]-thymidine between days 1 and 3. Uptake of [^3^H]-thymidine into hepatocyte DNA increased after EGF or HGF treatment in all rats, but DRH.F344-*Drh1* and DRH hepatocytes exhibited approximately 50–70% of the uptake observed in Donryu and F344 hepatocytes (Figures 6(a) and 6(b)).

### 3.4. Nuclear Size

**Figure 7:**
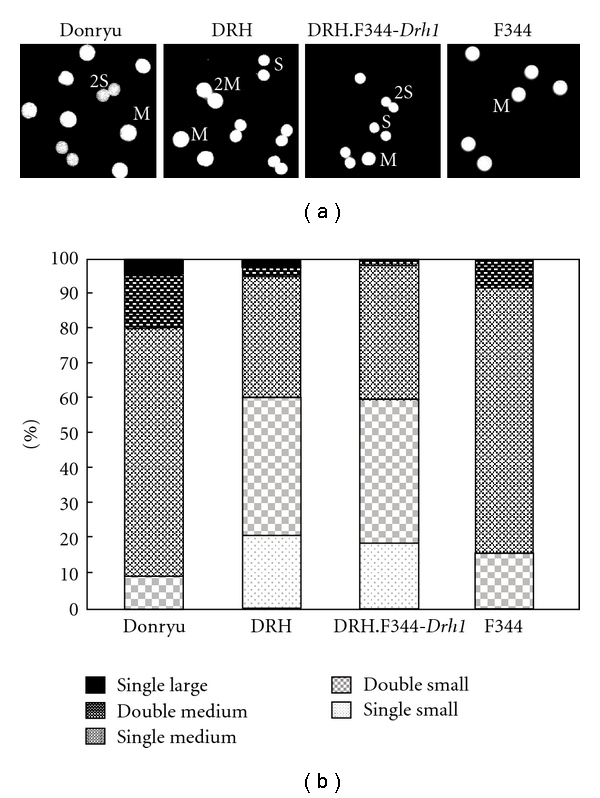
Nuclear size of hepatocytes. (a) DRH.F344-*Drh1* and DRH hepatocytes exhibited mainly single- (S) or double-small-sized nuclei (2S), while Donryu and F344 hepatocytes exhibited mainly single- (M) or double-medium-sized nuclei (2M). (b) Size distribution of hepatocyte nuclei. In DRH.F344-Drh1 and DRH rats, about 60% hepatocytes contain single- or double-small-sized nuclei, while other hepatocytes contain single-medium-sized nuclei. On the other hand, in Donryu and F344 rats, the majority of hepatocytes contain single- or double-medium-sized nuclei.

The DRH hepatocytes are smaller in size than Donryu hepatocytes [15]. We therefore compared the nuclear sizes of Donryu, DRH, DRH.F344-*Drh1*, and F344 hepatocytes collected from 8-week-old rats. When the DAPI-stained nuclear images of hepatocytes cultured for 2 h on cover glass were analyzed by Image J software, the nuclear size could be categorized into small, medium, and large classes (data not shown). Approximately 60% of DRH.F344-*Drh1* and DRH hepatocytes had single- or double-small-sized nuclei, while the remainder had single-medium-sized nuclei (Figures 7(a) and 7(b)). By contrast, 10–15% of Donryu and F344 hepatocytes had double-small-sized nuclei, while the remaining 85–90% had single- or double-medium-sized nuclei (Figures 7(a) and 7(b)). 

## 4. Discussion

 In DRH rats, two major genetic loci, *Drh1* on chromosome 1 and *Drh2* on chromosome 4, have been demonstrated to have strong effects on the number and size of preneoplastic hepatocytic lesions during hepatocarcinogenesis [5, 6]. DRH hepatocytes, on the other hand, have unique properties, including low levels of proliferation after LN treatment [4, 9], resistance to hepatotoxic chemicals [8], small cell size [15] and low levels of p38 and JNK activation *in vitro *[15]. If some of these properties are linked to the genetic loci, then the properties might be related to the mechanism(s) of hepatocarcinogenesis resistance in DRH rats. By comparing DRH.F344-*Drh1* hepatocytes with Donryu, DRH, and F344 hepatocytes, we demonstrated that low levels of proliferation and low levels of p38 dephosphorylation after LN treatment and resistance to hepatotoxic chemicals in DRH hepatocytes are linked to the *Drh1* locus, while low levels of proliferation *in vitro* and small nuclear size are not linked to this locus (Table 1). 

 It has been suggested that the hepatocyte proliferation induced by LN is at least partly due to the soluble factors generated by activated Kupffer cells [11, 12]. After LN treatment, the mRNA levels of TNF-*α*, IL-6, and Cox2, which are expressed in activated Kupffer cells [23, 24], were increased, and NF*κ*B and Stat3, which are transcription factors that can be activated, respectively, by TNF-*α* and IL-6 to play important roles in hepatocyte proliferation [19], were activated. However, there was no remarkable difference between DRH, Donryu, and DRH.F344-*Drh1* livers regarding cytokine and Cox2 mRNA expression and NF*κ*B or Stat3 activation after LN treatment, suggesting that the low level of proliferation of DRH hepatocytes induced by LN might not be due to the lack of factors that mediate hepatocyte proliferation; instead, they might be dependent on the inherent properties of DRH hepatocytes. 

 It has been reported that, although p38 is hyperphosphorylated in normal livers, it becomes hypophosphorylated after treatment with chemicals such as CCl_4_, d-galactosamine, and thioacetamide [20–22] and that p38 hypophosphorylation is mediated by activation of the phosphatase MKP-1 [20]. In the present study, we demonstrated that p38 dephosphorylation also occurred after LN treatment, an effect that was dependent on the *Drh1* locus. Although the mechanism of p38 dephosphorylation induced by LN is unknown, this phenomenon may be related to the low level of proliferation of DRH hepatocytes induced by LN and to hepatocarcinogenesis resistance in DRH rats.

 The resistance of DRH hepatocytes to DEN and CCl_4_ was also linked to the *Drh1* locus. Liu et al. [9] reported that when a 3′-Me-DAB diet was continuously provided, 17.9%, 11.3%, and 5.7% of the total liver tissues were made up of fibrotic areas in F344, DRH.F344-*Drh1*, and DRH rats, respectively, suggesting that the *Drh1* locus may also be related to resistance to chronic hepatic injury induced by 3′-Me-DAB. Hepatic injury of the liver tissue is considered important for the selective growth of preneoplastic hepatocytes during hepatocarcinogenesis [25]. In fact, transplanted DRH preneoplastic hepatocytes proliferate at high rates in Donryu livers treated with 2-AAF/PH, while transplanted Donryu hepatocytes proliferate at low rates in DRH livers treated with the same regimen [8]. Therefore, the tissue environment of the DRH liver may be less effective for the selective growth of preneoplastic hepatocytes during hepatocarcinogen treatment, which may be one of the mechanisms of hepatocarcinogenesis resistance in DRH rats. 

 The low level of proliferation of DRH hepatocytes *in vitro* is not linked to the *Drh1* locus. Hepatocyte proliferation *in vitro* is associated with complex changes, including the loss of hepatocyte-specific gene expression (e.g., albumin and cytochrome p4502A1), the gain of bile duct epithelium-specific gene expression (e.g., cytokeratin 19), and the activation of specific transcription factors (e.g., AP1 and NF*κ*B) [17]. In addition, the signals mediated by the extracellular matrix influence proliferation and gene expression in hepatocytes *in vitro *[17, 26–28]. Although the basis for the low level of proliferation of DRH hepatocytes *in vitro* remains to be investigated, DRH hepatocytes may have inherently low responsiveness to hepatocyte growth factors. Because the proliferation of preneoplastic hepatocytes is considered to be dependent on extracellular signals rather than on autonomous growth [25], the low level of proliferation of DRH hepatocytes *in vitro* may be related to the small preneoplastic hepatocytic lesions present in DRH livers during hepatocarcinogenesis.

 The small nuclear size of DRH hepatocytes was also not linked to the* Drh1* locus. Although hepatocytes have single-diploid nuclei during development, double-nuclear diploid and single- or double-nuclear tetraploid cells constitute the main populations in the adult liver [29]. Double-nuclear cells may be generated by a lack of cytokinesis after DNA synthesis, while tetraploid cells may be generated by a lack of mitosis after DNA synthesis in diploid cells [29]. On the other hand, polyploid hepatocytes increase in number under various pathophysiological conditions, including liver regeneration after partial hepatectomy [30], exposure to hepatotoxic chemicals such as retrorsine [31, 32], and metabolic defects associated with copper or iron deposition [33, 34]. Moreover, polyploid hepatocytes have been shown to express senescence phenotypes [35], including increased expression of p21 and *β*-galactosidase, which has been demonstrated in senescent cells [36, 37], low replication capacity, and high levels of apoptosis [35]. It is possible that suppressed or delayed tetraploidization in DRH hepatocytes reflects a diminished senescence phenotype, which may be linked to hepatocarcinogenesis resistance in DRH rats.

 In summary, DRH hepatocytes possess unique properties, some of which are linked to the *Drh1* locus. However, it remains to be investigated whether other properties are linked to the *Drh2* locus. Identification of the genes present in these loci may contribute to the clarification of the mechanism(s) and prevention of hepatocarcinogenesis. 

##  Conflict of Interests

The authors declare no conflict of interests. 

## Figures and Tables

**Table 1:** Comparison of hepatocytes from Donryu, DRH, DRH.F344-Drh1, and F344 rats.

	Donryu	DRH	DRH.F344-*Drh1 *	F344
Proliferation by LN	High	Low*	High*	High
P38 dephosphorylation	High	Low*	High*	High
Susceptibility to DEN and CCl_4_	High	Low*	High*	High^†^
Proliferation by GF	High	Low**	Low**	High
Nuclear size	Large	Small**	Small**	Large

*Phenotype different between DRH and DRH.F344-*Drh1*. **Phenotype similar between DRH and DRH.F344-*Drh1*. ^†^High to DEN as Donryu, but lower to CCl_4_ than Donryu.

## References

[1] Yoshimoto F, Masuda S, Higashi T (1985). Comparison of drug-metabolizing activities in the livers of carcinogen-sensitive parent rats and carcinogen-resistant descendants. *Cancer Research*.

[2] Higashi T, Fukui R, Sekiyama M, Yoshimoto F, Tateishi N, Sakamoto Y (1986). Decreased induction of *γ*-glutamyltransferase activity by 3′-methyl-4-dimethylaminoazobenzene in the liver of rats given carcinogen-containing diet for several generations. *Japanese Journal of Cancer Research*.

[3] Higashi K, Denda A, Higashi T, Hiai H (2004). Genetic resistance to chemical hepatocarcinogenesis in the DRH rat strain. *Comparative Medicine*.

[4] Denda A, Kitayama W, Konishi Y (1999). Genetic properties for the suppression of development of putative preneoplastic glutathione S-transferase placental form-positive foci in the liver of carcinogen-resistant DRH strain rats. *Cancer Letters*.

[5] Zeng ZZ, Higashi S, Kitayama W (2000). Genetic resistance to chemical carcinogen-induced preneoplastic hepatic lesions in DRH strain rats. *Cancer Research*.

[6] Yan Y, Zeng ZZ, Higashi S (2002). Resistance of DRH strain rats to chemical carcinogenesis of liver: genetic analysis of later progression stage. *Carcinogenesis*.

[7] Solt D, Farber E (1976). New principle for the analysis of chemical carcinogenesis. *Nature*.

[8] Imai K, Yamamoto M, Tanaka H (2007). Low selection of preneoplastic hepatocytes after treatment with the 2-acetylaminofluorene diet-partial hepatectomy regimen in the liver of hepatocarcinogenesis-resistant DRH strain rats. *Oncology Reports*.

[9] Liu H, Higashi K, Hiai H (2005). Role of resistant *Drh1* locus in chemical carcinogen-induced hepatocarcinogenesis in rats: analysis with a speed congenic strain. *Cancer Science*.

[10] Columbano A, Ledda GM, Sirigu P, Perra T, Pani P (1983). Liver cell proliferation induced by a single dose of lead nitrate. *American Journal of Pathology*.

[11] Columbano A, Shinozuka H (1996). Liver regeneration versus direct hyperplasia. *The FASEB Journal*.

[12] Shinozuka H, Ohmura T, Katyal SL, Zedda AI, Ledda-Columbano GM, Columbano A (1996). Possible roles of nonparenchymal cells in hepatocyte proliferation induced by lead nitrate and by tumor necrosis factor *α*. *Hepatology*.

[13] David SS, O’Shea VL, Kundu S (2007). Base-excision repair of oxidative DNA damage. *Nature*.

[14] Livneh Z (2006). Keeping mammalian mutation load in check: regulation of the activity of error-prone DNA polymerases by p53 and p21. *Cell Cycle*.

[15] Honmo S, Ozaki A, Yamamoto M (2007). Low p38 MAPK and JNK activation in cultured hepatocytes of DRH rats; a strain highly resistant to hepatocarcinogenesis. *Molecular Carcinogenesis*.

[16] Seglen PO (1976). Preparation of isolated rat liver cells. *Methods in Cell Biology*.

[17] Block GD, Locker J, Bowen WC (1996). Population expansion, clonal growth, and specific differentiation patterns in primary cultures of hepatocytes induced by HGF/SF, EGF and TGF*α* in a chemically defined (HGM) medium. *Journal of Cell Biology*.

[18] Rudnick DA, Perlmutter DH, Muglia LJ (2001). Prostaglandins are required for CREB activation and cellular proliferation during liver regeneration. *Proceedings of the National Academy of Sciences of the United States of America*.

[19] Grivennikov SI, Greten FR, Karin M (2010). Immunity, inflammation, and cancer. *Cell*.

[20] Mendelson KG, Contois LR, Tevosian SG, Davis RJ, Paulson KE (1996). Independent regulation of JNK/p38 mitogen-activated protein kinases by metabolic oxidative stress in the liver. *Proceedings of the National Academy of Sciences of the United States of America*.

[21] Nishioka H, Kishioka T, Iida C, Fujii K, Ichi I, Kojo S (2006). Activation of mitogen activated protein kinase (MAPK) during D-galactosamine intoxication in the rat liver. *Bioorganic and Medicinal Chemistry Letters*.

[22] Kishioka T, Iida C, Fujii K (2007). Effect of dimethyl sulphoxide on oxidative stress, activation of mitogen activated protein kinase and necrosis caused by thioacetamide in the rat liver. *European Journal of Pharmacology*.

[23] Wisse E, Braet F, Luo D (1996). Structure and function of sinusoidal lining cells in the liver. *Toxicologic Pathology*.

[24] Kmieć Z (2001). Cooperation of liver cells in health and disease. *Advances in Anatomy, Embryology, and Cell Biology*.

[25] Ogawa K (2009). Molecular pathology of early stage chemically induced hepatocarcinogenesis. *Pathology International*.

[26] Sérandour AL, Loyer P, Garnier D (2005). TNF*α*-mediated extracellular matrix remodeling is required for multiple division cycles in rat hepatocytes. *Hepatology*.

[27] Fassett J, Tobolt D, Hansen LK (2006). Type I collagen structure regulates cell morphology and EGF signaling in primary rat hepatocytes through cAMP-dependent protein kinase A. *Molecular Biology of the Cell*.

[28] Kim SH, Kim JH, Akaike T (2003). Regulation of cell adhesion signaling by synthetic glycopolymer matrix in primary cultured hepatocyte. *FEBS Letters*.

[29] Gupta S (2000). Hepatic polyploidy and liver growth control. *Seminars in Cancer Biology*.

[30] Brodsky W, Uryvaeva IV (1977). Cell polyploidy: its relation to tissue growth and function. *International Review of Cytology*.

[31] Torres S, Diaz BP, Cabrera JJ, Díaz-Chico JC, Díaz-Chico BN, López-Guerra A (1999). Thyroid hormone regulation of rat hepatocyte proliferation and polyploidization. *American Journal of Physiology—Gastrointestinal and Liver Physiology*.

[32] Oren R, Dabeva MD, Karnezis AN (1999). Role of thyroid hormone in stimulating liver repopulation in the rat by transplanted hepatocytes. *Hepatology*.

[33] Terada K, Sugiyama T (1999). The Long-Evans Cinnamon rat: an animal model for Wilson’s disease. *Pediatrics International*.

[34] Kato J, Kobune M, Kohgo Y (1996). Hepatic iron deprivation prevents spontaneous development of fulminant hepatitis and liver cancer in Long-Evans Cinnamon rats. *Journal of Clinical Investigation*.

[35] Sigal SH, Rajvanshi P, Gorla GR (1999). Partial hepatectomy-induced polyploidy attenuates hepatocyte replication and activates cell aging events. *American Journal of Physiology —Gastrointestinal and Liver Physiology*.

[36] Dimri GP, Lee X, Basile G (1995). A biomarker that identifies senescent human cells in culture and in aging skin *in vivo*. *Proceedings of the National Academy of Sciences of the United States of America*.

[37] El-Deiry WS, Harper JW, O’Connor PM (1994). WAF1/CIP1 is induced in p53-mediated G1 arrest and apoptosis. *Cancer Research*.

